# Cholera Toxin B Subunit Shows Transneuronal Tracing after Injection in an Injured Sciatic Nerve

**DOI:** 10.1371/journal.pone.0144030

**Published:** 2015-12-07

**Authors:** Bi-Qin Lai, Xue-Chen Qiu, Ke Zhang, Rong-Yi Zhang, Hui Jin, Ge Li, Hui-Yong Shen, Jin-Lang Wu, Eng-Ang Ling, Yuan-Shan Zeng

**Affiliations:** 1 Key Laboratory for Stem Cells and Tissue Engineering (Sun Yat-sen University), Ministry of Education, Guangzhou, 510080, China; 2 Department of Histology and Embryology, Zhongshan School of Medicine, Sun Yat-sen University, Guangzhou, 510080, China; 3 Institute of Spinal Cord Injury, Sun Yat-sen University, Guangzhou, 510120, China; 4 Co-innovation Center of Neuroregeneration, Nantong University, Nantong, 226001, China; 5 Department of Orthopedics of Sun Yat-sen Memorial Hospital, Sun Yat-sen University, Guangzhou, 510120, China; 6 Department of Electron Microscope, Zhongshan School of Medicine, Sun Yat-sen University, Guangzhou, 510080, China; 7 Department of Anatomy, Yong Loo Lin School of Medicine, National University of Singapore, Singapore, 117597, Singapore; 8 Guangdong Provincial Key Laboratory of Brain Function and Disease, Zhongshan School of Medicine, Sun Yat-sen University, Guangzhou, 510080, China; Hertie Institute for Clinical Brain Research, University of Tuebingen., GERMANY

## Abstract

Cholera toxin B subunit (CTB) has been extensively used in the past for monosynaptic mapping. For decades, it was thought to lack the ability of transneuronal tracing. In order to investigate whether biotin conjugates of CTB (b-CTB) would pass through transneurons in the rat spinal cord, it was injected into the crushed left sciatic nerve. For experimental control, the first order afferent neuronal projections were defined by retrograde transport of fluorogold (FG, a non-transneuronal labeling marker as an experimental control) injected into the crushed right sciatic nerve in the same rat. Neurons containing b-CTB or FG were observed in the dorsal root ganglia (DRG) at the L4-L6 levels ipsilateral to the tracer injection. In the spinal cord, b-CTB labeled neurons were distributed in all laminae ipsilaterally between C7 and S1 segments, but labeling of neurons at the cervical segment was abolished when the T10 segment was transected completely. The interneurons, distributed in the intermediate gray matter and identified as gamma-aminobutyric acid-ergic (GABAergic), were labeled by b-CTB. In contrast, FG labeling was confined to the ventral horn neurons at L4-L6 spinal segments ipsilateral to the injection. b-CTB immunoreactivity remained to be restricted to the soma of neurons and often appeared as irregular patches detected by light and electron microscopy. Detection of monosialoganglioside (GM1) in b-CTB labeled neurons suggests that GM1 ganglioside may specifically enhance the uptake and transneuronal passage of b-CTB, thus supporting the notion that it may be used as a novel transneuronal tracer.

## Introduction

For decades, development of new axonal tract tracing and labeling cell techniques has proven to be invaluable in charting the complex interconnections of the central nervous system (CNS) [1]. The commonly used tracers, such as enzymes [2], plant lectins [3] and virus have posed many interpretation problems [4] because of the variability in the quality of uptake and transport of these tracers as well as their tendency to spread in the tissue and capacity to produce local necrosis.

The plant enzyme horseradish peroxidase (HRP) was the first retrograde neuroanatomical tracer to be used [5]. Free HRP exhibits no specific affinity for the cell surface of neurons, and its use to label primary afferent terminals has limitations due to the lack of anterograde transport [6]. An alternative method using cytotoxic lectins, such as wheat-germ agglutinin (WGA) was then introduced. Active uptake is one advantage of the lectin. When WGA was conjugated to HRP (WGA-HRP), it greatly enhanced the uptake and transport of this molecule, and showed a much higher sensitivity than HRP alone [7]. WGA-HRP may also be used as a transneuronal tracer, permitting studies of multisynaptic pathways. But WGA-HRP shows a markedly reduced sensitivity in its passage across the synapses [1]. In contrast, neurotropic viruses replicate for signal amplification and traverse multi-synaptic pathways [8]. Under favorable conditions, neurotropic virus can be used to localize the first, second, and sometimes third order neurons that regulate a specific target. It does, however, have a number of disadvantages such as death of infected cells; other factors that are likely to affect the tracer labeling include species, age of animals, specific neural pathways, transmissibility and virulence, all of which may render the virus tracers unsuitable [9]. In view of the above, it would be desirable to search for a non-virus tracer which has potential value as a specific transneuronal marker that bears sensitivity and light stable in labeled neurons.

From the standpoint of using non-virus tracer as CNS transneuronal marker, use of bacterial toxins was critical [10]. Bacterial toxins are characterized by their high affinity for specific sugars. After binding to the cell membrane of neurons, they are internalized and transported within the dendrites and axon. The cholera toxin B subunit (CTB) binds to cellular surfaces via its receptor monosialoanglioside (GM1) [11]. As a conventional bacterial toxin for neuronal tracing, CTB enters the axons or dendrites by active uptake via nerve terminals, or through severed axons [12]. Biotin conjugates of CTB (b-CTB) may greatly enhance the uptake, transport and sensitivity of CTB [13]. It is possible that b-CTB may enhance uptake but inhibit transneuronal labeling. The issue of whether b-CTB is a transneuronal marker has remained to be ascertained. Here, we have tested the hypothesis by injecting b-CTB into the rat sciatic nerve to determine if it would result in transneuronal labeling in the spinal cord. For experimental control, the first order afferent neuronal projections were defined by fluorogold (FG). FG (yellow-golden dye) is a typical non-transneuronal retrograde tracer. Following its uptake by injured axonal profiles and terminals, the dye accumulates in vesicles and is transported in a retrograde direction, and often gives a granular appearance to the labelled cell somata [1]. However, FG may have toxicity to injected nerve tissue and induce motor neuron deficits. Therefore, it is not suitable for long-term labelling.

## Materials and Methods

### Surgical Procedures and Biotin-CTB Injection into Sciatic Nerve

All surgical procedures were performed under the sterile condition in a designated animal surgery area. Adult female Sprague-Dawley (SD) rats (*n* = 5, 220–250 g, supplied by the Experimental Animal Center of Sun Yat-sen University, S1 License and S2 License) were anesthetized with 1% pentobarbital sodium (40 mg/kg, i.p.). A longitudinal incision was made in the skin over the upper posterior part of the left thigh and the gluteal region. The gluteus maximus muscle was then separated with a pair of iris scissors along the direction of its fibres to expose the sciatic nerve. To minimize animal suffering, 2% lidocaine was applied with a cotton swab. The left sciatic nerve (about 1 cm distal to the sciatic notch) was crushed with a 2 mm width blunt forceps for 60 s before the injection of b-CTB to facilitate maximal tracer contact with nerve fibers. Complete crush was confirmed by the presence of a translucent band across the nerve. Under the guidance of a Leica MZ6 dissecting stereomicroscope (Leica Microsystems, Inc., Wetzlar, Germany), the needle tip of a glass micropipette was inserted into the nerve just distal to the site of crush. After this, a total of 2.5 μl of 1% biotin-CTB (Molecular Probes, C-34779) was slowly injected over approximately 20 s. The needle tip was left in place for 3 to 4 min and was removed slowly over 15 to 20 s to prevent leakage of injection solution. Following injection, the surface of the injection site was washed with a saline soaked cotton swab, and the nerve was ligated between the sites of inoculation and where the micropipette had penetrated the epineurium; the wound was then closed with sutures. Along with the above, 1 μl of 3% FG (Fluorochrome, Englewood, CO) was injected into the right sciatic nerve in the same manner.

In addition, 5 rats subjected to a complete transection of the spinal at T10 level and allowed to survive for 2 months after surgery as described in our previous work [14] were given the same injection of both tracers. Rats were prophylactically treated with ampicillin, 100 mg/kg (Fort Dodge, IA). All experimental protocols and animal handling procedures were approved by the Animal Care and Use Committee of Sun Yat-sen University, and were consistent with the National Institutes of Health Guide for the Care and Use of Laboratory Animals.

### Perfusion and Tissue Preparation

Animals were sacrificed 7 days after b-CTB and FG injections. All rats were deeply anesthetized with 1% pentobarbital sodium (50 mg/kg, i.p.) and systemically perfused with physiological saline containing 0.002% NaNO_2_ and 0.002% heparin, followed with 4% paraformaldehyde. After perfusion, the L3-S1 levels of dorsal root ganglia (DRG), and spinal cord were removed and post-fixed overnight in the same fixative. Tissue was then placed in 30% sucrose/phosphate buffered saline (PBS). Transverse sections of selected spinal cord segments were cut at 25 μm thickness; every 5th sections of the spinal cord were collected. The L3-S1 DRG were sectioned at 25 μm thickness. All sections were stored at -20°C until further processing.

### Immunofluorescence Staining

Specific proteins were determined using immunofluorescence staining as described in our previous study [14,15]. Briefly, sections were blocked with 10% normal goat serum and 0.3% Triton X-100 in PBS (phosphate-buffered saline 0.1 M, pH 7.4) for 1 h at 37°C. After blocking, sections were incubated with primary antibodies mixed in 0.3% Triton X-100 overnight at 4°C, followed by incubation with secondary antibodies and Hochest33342 (Hoe). For examination of b-CTB-labeled neurons, b-CTB staining was performed at 37°C with avidin-cy3 (1:300) for 1h. The slides were then examined under a fluorescence microscope. A summary of antibodies used is provided in Table 1.

**Table 1:** Primary and secondary antibodies used.

Antibodies	Species	Type	Dilution	Source (Catalog)
Green fluorescent protein (GFP)	Rabbit	Polyclonal IgG	1:500	Sigma, St. Louis, USA (G1544)
Microtubule-associated protein 2 (MAP2)	Mouse	Monoclonal IgG	1:1000	Sigma, St. Louis, USA (M1406)
Ganglioside GM1	Rabbit	Polyclonal IgG	1:100	Abcame (ab23943)
Alexa 647 conjuncted anti rabbit secondary antibody	Goat	Polyclonal IgG	1:500	Jackson ImmunoResearch, West Grove, USA (100699)
Alexa 488 conjuncted anti rabbit secondary antibody	Goat	Polyclonal IgG	1:300	Molecular Probes (A-11034)
Alexa 488 conjuncted anti mouse secondary antibody	Goat	Polyclonal IgG	1:300	Molecular Probes (A-11029)
Dylight 405 anti mouse secondary antibody	Goat	Polyclonal IgG	1:200	Jackson ImmunoResearch, West Grove, USA (93828)
Ionized calcium binding adaptor molecule 1(IBA-1)	Rabbit	Polyclonal IgG	1:500	Wako, Japan (019–19741)
Glutamate decarboxylase (GAD67)	Rabbit	Polyclonal IgG	1:300	Boster (BA0603)
Avidin-cy3			1:300	Boster, Wuhan, China (BA1037)
Avidin-HRP			1:1500	Boster, Wuhan, China (BA1081)

### Histochemical staining

Peroxidase staining was performed according to the avidin–biotin peroxidase method. Briefly, after sectioning and rinsing in PBS, the sections were pretreated with 0.3% H_2_O_2_ for l0 min, rinsed (PBS, 3 × 5 min), incubated with 2.5% bovine serum albumin and 0.3% Triton X-100 in PBS for 0.5 h, and then rinsed again (PBS, 3 × 5 min). After this, the sections were incubated in avidin-HRP (1:1,500) for 1 h, rinsed (PBS, 3 × 5 min), and then reacted with diaminobenzidine (0.05%) as a chromogenic agent, with reaction sustained for 10 min at room temperature. After the reactions, the sections were dehydrated, cleared, and covered with coverslips. Slides were then examined under a laboratory light microscope.

### Ultrastructural Observations

For immunoelectron microscopy (IEM), rats were transcardially perfused with 0.1 mol/L of sodium phosphate buffer containing 187.5 units/100 ml of heparin, followed by perfusion with 4% paraformaldehyde, 0.1% glutaraldehyde, and 15% saturated picric acid. Dissected spinal cord was postfixed overnight at 4°C in fresh fixative and subsequently cut into 50 μm thick sagittal sections on a vibratome. To improve the penetration of antibodies, vibratome sections were transferred into cryprotectant solution containing 25% sucrose and 10% glycerol in 0.1 M PBS overnight at 4°C, followed by a quick freeze-thaw in liquid nitrogen three times. After washing with PBS, the sections were treated for 1 h with 20% goat serum (Tris buffer, pH 7.4) to block nonspecific binding of the antibody. Sections were first incubated with primary antibodies in 2% normal goat serum solution at 4°C for 24 h, then incubated with secondary antibodies overnight at 4°C, and postfixed in 1% glutaraldehyde for 10 min. The sections were detected by SABC-DAB Kit (Vectastain PK6102) and silver enhanced with HQ silver Kit (NanoProbe 2012, Yaphank, NY), osmicated, dehydrated, and embedded in Epon. Epon blocks were sectioned and examined under the electron microscope (Philips CM 10, Eindhoven, Holland).

### Morphological Quantification

For quantification of b-CTB positive cells, a total of 5 sections cut respectively through the L4, T10 and C7 spinal cord segments per rat were chosen (*n* = 5). All b-CTB positive cells from each of the sections were enumerated. The average neuronal number per section for each rat was used for respective spinal cord segment statistics. For quantification of FG and b-CTB, retrogradely double labeled with ChAT or GAD67 at the C7/L4 spinal segment, 100 ChAT or GAD67 positive cells/each rat (*n* = 5) were detected.

### Statistical Analysis

All statistical analyses were performed using the statistical software SPSS13.0. Data were reported as means ± standard deviations (SD). When three sets of data were compared, one-way ANOVA with a LSD-t (equal variance assumed) or Dunnett's T3 (equal variance not assumed) was performed. A statistically significant difference was accepted at *P* < 0.05.

## Results

### FG and b-CTB Labeling in Dorsal Root Ganglia (DRG)

**Fig 1:**
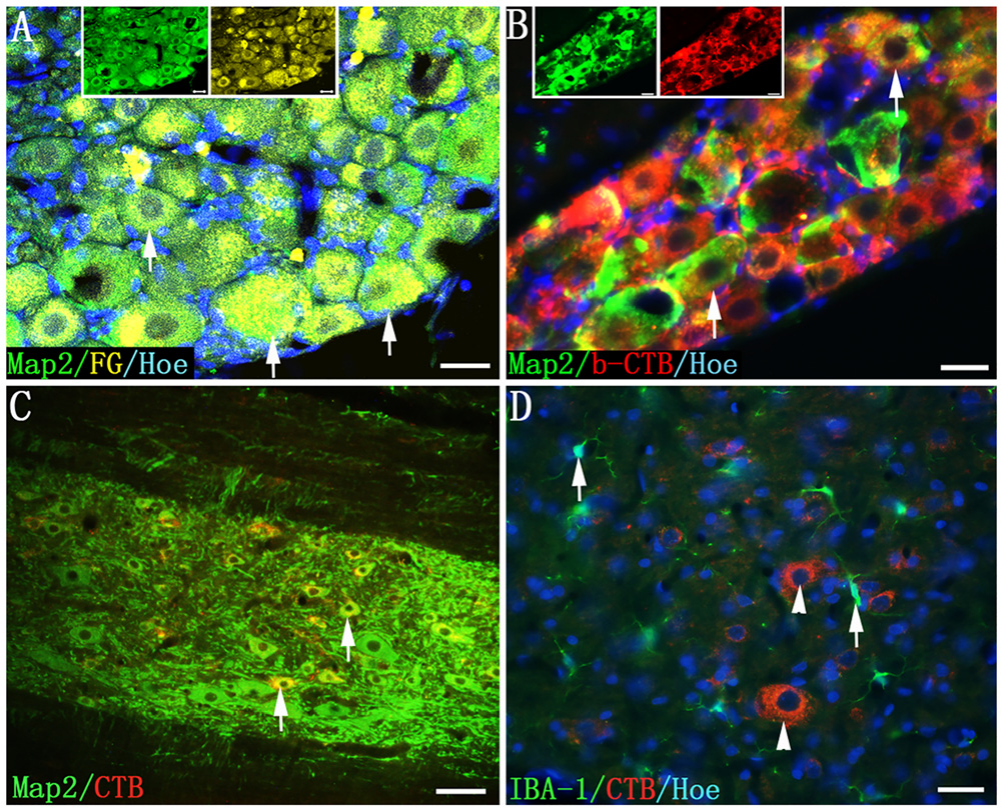
Retrogradely labeled neurons following FG and b-CTB injection into the sciatic nerve. (A) In L4 DRG, FG-labeled neurons (Map2, green) of various sizes emit whitish-yellow fluorescence in the cytoplasm (arrows); (B) Most of the b-CTB reaction products are contained within the neuronal somata in a L4 DRG, and b-CTB (red) and Map2 (green) dually-labeled neurons appear yellow (arrows); (C) b-CTB positive products are confined within the Map2 positive cell somata in T9 spinal segment (arrows); (D) b-CTB (red) is not phagocytosed by IBA-1 positive cells (green, arrows). Scale bars = 20 μm in (A) and (B); 40 μm in (C) and (D).

At 7 days following unilateral injections of FG and b-CTB, respectively into the left and right sciatic nerve, retrogradely labeled cell bodies in L3-S1 DRGs were observed. In all animals, on the corresponding site injected with the respective tracer, L4 DRG were intensely labeled by FG (Fig 1A) and b-CTB (Fig 1B). Labeling was less evident in DRG at L6 level (data not shown). FG and CTB labeled cell profiles were rarely observed in the L3 DRG and were absent in S1 DRG (data not shown). FG and b-CTB immunoreactivity was most intense within microtubule associated protein 2 (Map2, a marker for postmitotic neurons) positive neurons in the L4 DRG. A majority of the neurons in the appropriate DRG were labeled (Fig 1A and 1B), suggesting that DRG had received successful injections of FG and b-CTB into the sciatic nerve.

### b-CTB Labeling of Neurons

To ascertain if b-CTB injected into crushed sciatic nerves would open up the possibility of phagocytosis-dependent cell labeling, or leakage of the tracer in the spinal cord tissue as has been reported for FG or lectin [16,17]. b-CTB and Map2 double labeling was performed. The results showed that b-CTB reaction products were detected only in the soma of Map2 positive neurons (Fig 1C). The non-neuronal cells were devoid of b-CTB immunoreactivity (Fig 1C) even in rats that were allowed to survive for 7 days after the injection of the tracer. Double labeling of b-CTB and ionized calcium binding adaptor molecule 1 (IBA-1, a marker of microglia/macrophage) that allows for direct and simultaneous visualization of both b-CTB labeled cells and microglia/macrophages showed that there was no nonspecific phagocytosis-dependent b-CTB labeling in the spinal cord (Fig 1D). Actually, we counted 500 IBA-1 positive cells derived from five b-CTB injected rats (100 cells/each rat) in L4 spinal segment; none of them showed nonspecific phagocytosis-dependent b-CTB labeling (data not shown).

### FG and CTB Labeling in Spinal Cord

**Fig 2:**
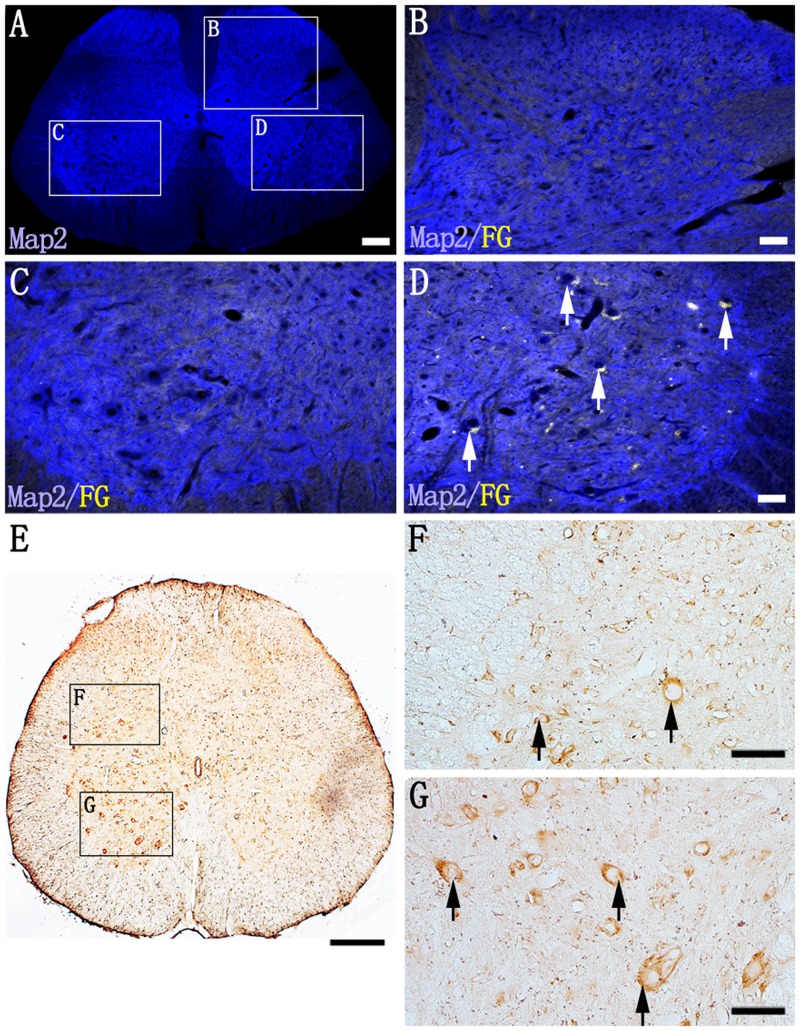
Comparison of labeling patterns of FG and b-CTB following injection of the tracers into the right and left sciatic nerves, respectively. (A) Showing an overview of a transverse section of L4 spinal segment. Map2 staining (blue) helps to define the grey matter of spinal cord. The three boxed areas (B, C, D) in (A) are magnified in images (B), (C) and (D), respectively. Note in (B), FG-labeled neurons are absent in the interneuron region i.e. areas deep to the dorsal horn. In (C), FG-labeled neurons are absent in the left ventral horn; in (D), Map2/FG double labeled neurons (arrows) with their contents of FG fluorescence are readily identified in the right ventral horn. In (E), at the C7 spinal segment, after injection of b-CTB (into the left sciatic nerve), labeled neurons are widely distributed in the ipsilateral spinal segments. b-CTB-labeled neurons are not detected in the contralateral ventral horn. b-CTB labeled neurons are distributed in interneuron region (F, arrows, boxed area in (E)), and motoneuron region (G, arrows, boxed area in (E)). Scale bars = 200 μm in (A) and (E); 50 μm in (B), (C), (F) and (G).

**Fig 3:**
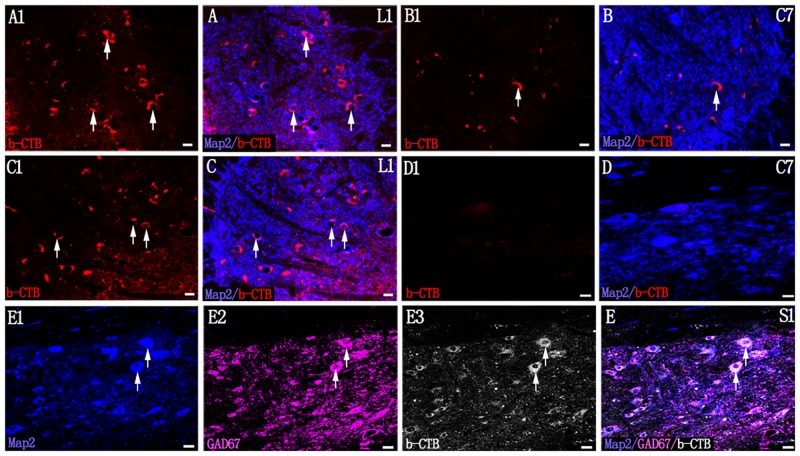
Evidences supporting transneuronal labeling of b-CTB. (A1) and (A) In the uninjured spinal cord, b-CTB-labeled neuronal somata (red and blue, arrows) are detected at L1 spinal segment. (B1) and (B) b-CTB-labeled neuronal soma (red and blue, arrow) at C7 segment of the uninjured spinal cord. (C1) and (C) b-CTB (C1, red, arrows) -labeled neuronal somata (C, blue, arrows) at L1 spinal segment below the transection of the spinal cord at T10 level. (D1) and (D) b-CTB-labeled neuronal somata (blue) are absent at C7 spinal segment above the transection site of the spinal cord at T10. (E)-(E3) Showing b-CTB-labeled somata of interneurons (arrows) in S1 segment of the uninjured spinal cord when stained with anti-Map2 (E1, blue, arrows), anti-GAD67 (E2, magenta, arrows) and b-CTB (E3, grey, arrows). Scale bars = 40 μm in (A1-E).

After FG injection into the right sciatic nerve, most FG positive neurons were confined to the ipsilateral ventral horn of L4 spinal segments, and they were mainly the larger motor neurons (Fig 2A–2D; S1 Fig). Seventy-seven (77%) of ChAT positive neurons at L4 ventral horns were co-labeled with FG (Table 2; S1 Fig). FG labeled neurons were absent in the upper L3 spinal segments (data not shown). However, as distinct from FG injection (S2 Fig), b-CTB-labeled cell clusters were detected rostrally up to the C7 spinal segment (Fig 2E). The majority of b-CTB labeled neurons were distributed in the ipsilateral deep dorsal horn (Fig 2F) and ventral horn (Fig 2G). At C7 segment, 16% of ChAT labeled cells were b-CTB positive and 18.2% GAD67 labeled cells were b-CTB positive (Table 2; S3 Fig). While, at L4 segment, more ChAT or GAD67 positive cells were co-labeled with b-CTB (Table 2; S1 Fig). b-CTB reaction products were localized in the neuronal soma of L1 and C7 spinal segments (Fig 3A1 and 3A, 3B1 and 3B, 3C1 and 3C), but b-CTB labeling of neuronal processes was less evident. Following T10 spinal cord transection, b-CTB labeled neurons were absent at the cervical segment level (Fig 3D1 and 3D). GAD67 immunofluorescence staining confirmed that the b-CTB positive neurons in the intermediate gray matter of S1 spinal cord segment were gamma-aminobutyric acid-ergic (GABAergic) interneurons (Fig 3E1 and 3E).

**Table 2:** Comparison of number of FG and b-CTB positive cells stained doubly with ChAT or GAD67 in the C7 and L4 segments of spinal cord (mean ± SD, %).

Spinal segment	*n*	FG/ChAT	b-CTB/ChAT	FG/GAD67	b-CTB/GAD67
C7	5	0.00 ± 0.00	16.00 ± 1.58	0.00 ± 0.00	18.20 ± 1.48
L4	5	77.00 ± 2.74	74.60 ± 2.88	0.00 ± 0.00	72.80 ± 2.17

### Histological Features of b-CTB-labeled Neurons

**Fig 4:**
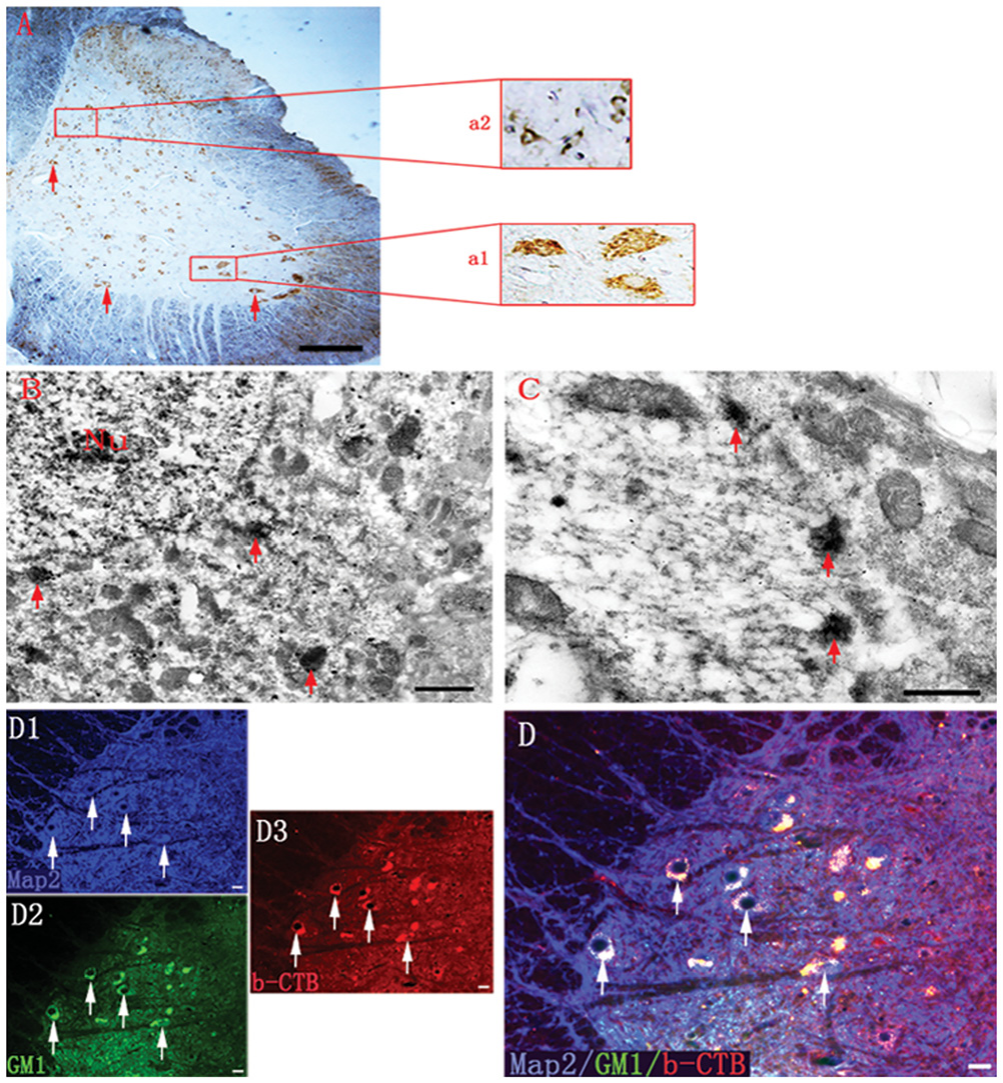
Localization of b-CTB reaction products and GM1 expression. (A) Transverse section at L3 spinal segment shows brown crystalline cytoplasmic staining of b-CTB-DAB reaction products in neuronal somata of various sizes in all laminae (arrows); (a1) Showing neurons containing granular b-CTB reaction products in ventral horn area; (a2) Showing neurons containing granular b-CTB reaction products in the Clarke’s nucleus. (B) Immunoelectron micrograph showing b-CTB in the T10 segment of spinal cord. Note b-CTB-labeled neuron displays some electron-dense reaction products (arrows) scattered in the soma; Nu = nucleus. (C) Localization of irregular patches of the electron-dense reaction products in the soma (arrows). (D1) Transverse section of the ventral horn of T10 segment showing Map2 positive neurons (arrows). (D2) These neurons overlap extensively with GM1 (arrows). (D3) GM1 positive neurons are also labeled by b-CTB (arrows). (D) A merged image of (D1-D3) displays that almost all b-CTB-labeled neurons are positive for GM1 (arrows). Scale bars = 80 μm in (A); 1 μm in (B); 500 nm in (C); 40 μm in (D1-D3, D).

b-CTB labeled neurons in the ventral horn and Clarke’s nucleus in L3 spinal segment exhibited some DAB brown aggregates localized mainly in the soma (Fig 4A). At the electron microscopic level, the DAB reaction products were visualized as irregular electron-dense patches scattered in the b-CTB positive soma. (Fig 4B and 4C).

### GM1 Ganglioside Expression in b-CTB Labeled Neurons

The expression of CTB receptor GM1 ganglioside (Fig 4D1 and 4D2) as well as intense b-CTB-labeling (Fig 4D3) was detected in the ventral horn neurons (Fig 4D). Since GM1 ganglioside was localized in highest concentration in the neuronal plasma membrane, receptor-mediated internalization and intracellular transport of b-CTB is deemed to be efficient [12].

### Segmental Distribution of b-CTB Labeled Neurons

**Fig 5:**
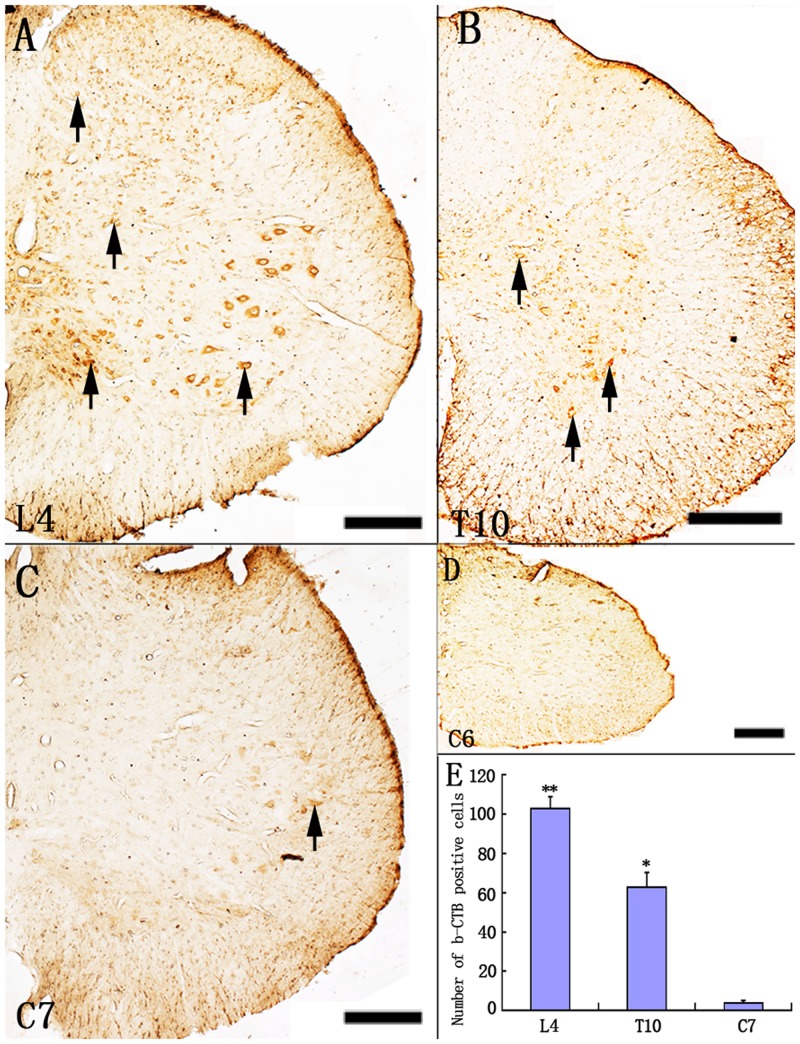
Distribution of b-CTB positive cells in different segments of spinal cord. Representative images show b-CTB positive cells (arrows) in the spinal cord segments at L4 (A), T10 (B), C7 (C) and C6 (D). Note that the central labeling for b-CTB extends up to the C7 segment, but absent in the C6 segment. Scale bars = 40 μm in (A-D). (E) Mean number of b-CTB positive cells is counted in the L4, T10 and C7 segments. Data are expressed as means ± SD (*n* = 5 rats). **P < 0.001, compared with T10 and C7; * *P* < 0.001, compared with C7.

After injection of b-CTB into the left sciatic nerve, b-CTB labeled cells were observed in the spinal cord extending between C7-S1 segments, with ipsilateral labeling being predominant. Many b-CTB positive neurons were detected in the lumbar segment of spinal cord. A lower incidence of b-CTB positive neurons was detected in the thoracic segment. They were haphazardly distributed in the cervical spinal cord, but did not extend beyond the C7 segment rostrally. We selected the L4, T10 C7 and C6 segments representing the lumbar, thoracic and cervical segments, respectively, for neuronal enumeration (Fig 5A–5D). No b-CTB positive neuron was observed in the C6 segment (Fig 5D). Cell counting showed more b-CTB positive neurons were detected in the L4 segment compared with the T10 and C7 segments (Fig 5E).

## Discussion

The main purpose of neural tract tracing is to chart anatomical connections within the nervous system [1]. Neurotropic viruses such as pseudorabies virus (PRV) are an extremely powerful neuroanatomical tool for synapse-dependent retrograde transneuronal studies of neuronal connectivity [8]. On the other hand, in some instances transneuronal transport of neurotropic viruses was not satisfactory indicating that some factors such as system, tropisms or species differences might affect the effectiveness of virus as a transsynaptic tracer [4,18]. To minimize or obviate the effects of undesirable factors that might affect the tracer uptake or transport, we have focused in this study on b-CTB and explored its potential use as a transneuronal tracer. If proven effective, b-CTB would facilitate a more reliable and accurate mapping of neural connectivity for a better understanding of the anatomical and functional organization of the nervous system.

It is unequivocal in this study that labeled L4 DRG neurons and L4 spinal cord ventral horn neurons are due to the retrograde intra-axonal transport of b-CTB and FG administered into the sciatic nerve. The results also indicate the possibility of transneuronal transport of b-CTB on entry through retrograde axonal transport into the spinal cord, because neither b-CTB leakage nor phagocytosis-dependent b-CTB labeling was observed. The regions showing evidence of transneuronal labeling include the presence of a group of b-CTB positive neurons in the deep dorsal horn (Fig 2G), intermediate grey matter GABAergic neurons (Fig 3E) and Clarke’s nucleus (Fig 4A) of the spinal cord. Occasional b-CTB labelled neurons were also observed on the contralateral side. On the contrary, FG as first order afferent tracer was only detected in the ventral horn motor neurons of ipsilateral L4-L6 spinal segments. The underlying mechanism for the above phenomena is not clear. Active uptake is one of the advantages of the CTB [19]. Since receptor-mediated internalization is very efficient, CTB is internalized at a much higher rate than HRP or FG [20], and lower concentration of b-CTB (1%) is suffice for labeling purpose. The adsorptive endocytosis of CTB is transported to the trans-most cisterns and tubules of the Golgi apparatus, as well as to lysosomes in cell bodies [21]. This may explain on why b-CTB, as detected by light and electron microscopy, produces a granular appearance in the cell somata, but is less evident in neuronal processes [22].

A striking observation in the present results was the intense localization of b-CTB in the deep dorsal horn, the column of Clarke and the intermediate grey matter of the spinal cord. These interneurons such as GABAergic neurons may receive inputs from ventral horn motor neurons or sciatic nerve afferent neurons, demonstrating the transneuronal passage of b-CTB by the existence of neuronal connections. A possible explanation for b-CTB labelling of interneurons would be that these cells were endowed with abundant plasma membrane receptors for transport of b-CTB. This is supported by monosialoanglioside (GM1) immunostaining in the b-CTB positive neurons. GM1 ganglioside is widely distributed in all tissues, but occurs in highest concentrations in the central nervous system (CNS) [23]. It is primarily located in the outer surface of the mammalian plasma membranes and in synaptic membranes of the CNS [24]. Transneuronal transport of b-CTB, may be enhanced by receptor-mediated internalization [23]. The b-CTB in combination with GM1 ganglioside was then transported in the cytoplasm via the endoplasmic reticulum and Golgi apparatus [11]. Subsequently, it is released either in a non-specific fashion by exocytosis into the extracellular space [25] where they may be phagocytosed by nearby neurons or by a specific transfer process to contiguous second order neurons that make synaptic contacts on the first order neurons [26].

Previous investigations had reported that CTB was incapable of being trans-synaptically transported [22]. On the other hand, we show here that b-CTB is a potential and specific transneuronal marker. This is supported by the labeling of interneurons in different laminae of the spinal cord extending from S1-C7 levels following b-CTB injection into the sciatic nerve. Remarkably, in rats subjected to a total transection of the spinal cord at T10 level, b-CTB labeled neurons were absent at the cervical level (C7). This suggests that the rostral transneuronal transport of b-CTB was interrupted by the cord transection that had damaged the axons. A possible explanation for the discrepancy between the present results and those of previous studies by others would be that the sciatic axon crush had led to *de novo* and increased expression of GM1 ganglioside in the injured neurons [12,27] as demonstrated in this study. The fact that GM1 ganglioside occurs in highest concentrations in plasma membranes renders b-CTB capable of being trans-synaptically transported [28]. Previous studies suggested that b-CTB may bypass the retrograde pathway by GM1 ganglioside-mediated transcytosis [29]. This process may enhance the sensitivity of b-CTB as a transneuronal tracer. We have used CTB conjugated to biotin that may increase the relative sensitivity compared with free CTB or CTB conjugated to fluorochromes. This is because the specific interactions between biotin and its receptors may facilitate a much higher cellular uptake for b-CTB delivery [13]. An additional advantage of b-CTB is that it can be visualized with an avidin-biotinylated procedure (avidin-biotin complex, ABC, method), followed by diaminobenzidine (DAB) or immunofluorescence reaction. The signal is amplified and with improved sensitivity, thus allowing double labelling that can be visualized at the light- and electron-microscopic levels.

Unlike other studies, which demonstrated that primary afferents from the sciatic nerve project predominantly to the ipsilateral gracile nucleus and several other brainstem nuclei [30], we did not find any b-CTB positive neurons in the brainstem. The b-CTB we used tended to remain in neuronal somata and it does not adequately reveal the detailed morphology of neuronal processes [1,31]. The b-CTB positive elements in the lower level of the cervical spinal cord were consistently less intense and extensive than in the lumbar level. The diminution in transneuronal b-CTB labeling as observed from S1 and C7 may be due to the progressive reduction in quantity of b-CTB transported across the neurons or the number of axon terminals available for b-CTB uptake; the length of the axon through which the tracer must be transported before it can contribute to transneuronal labeling is another consideration.

The retrograde, anterograde [32] and transneuronal transport of b-CTB makes it unsuitable for studies seeking to identify the accurate origin of neurons terminating at the application site. It is unable to replicate for signal amplification and travel multi-synaptic pathways like a viral tracer. But within a short distance, b-CTB tracing can at least provide an invaluable approach for investigating the structural and spatial relationship between first order neurons and its second order target neurons and perhaps even beyond this.

## Conclusions

We show here that injection of b-CTB into the sciatic nerve could label interneurons in the spinal cord. The present results suggest that the specific interactions between CTB and GM1 ganglioside as well as biotin and its receptors may be a mechanism of b-CTB to be transported trans-neuronally. Thus, b-CTB may be used as a new tracer for transneuronal labeling. More importantly, b-CTB can be visualized at the light- and electron-microscopic levels, which will further advance its application in tracing neural network connectivity.

## Supporting Information

S1 Fig Comparison between b-CTB and FG tracing at L4 spinal segment. (A) Showing a merged image of FG (A1, arrows, yellow)-labeled neurons stained doubly with ChAT (A2, arrows, blue). (B) Showing a merged image of b-CTB (B1, arrows, red) co-labeled with ChAT (B2, arrows, blue). (C) Showing a merged image of FG (C1)-labeled neurons to be absent when stained doubly with GAD67 (C2, arrows, blue). (D) Showing a merged image of b-CTB (D1, arrows, red) co-labeled with GAD67 (D2, arrows, blue). Scale bars = 20 μm.
(DOC)

S2 Fig C7 spinal segment for FG tracing. (A) Showing an overview of a transverse section of C7 spinal segment. Note the total absence of FG positive cells. (B) Map2-labeled staining (blue) helps to define the grey matter of spinal cord central area. (D) Showing a merged image of (A) and (B). Scale bars = 200 μm.
(DOC)

S3 Fig Interneuron versus motor neuron labeling at C7 spinal segment. (A) Showing a merged image of FG (A1)-labeled neurons to be absent when stained doubly with ChAT (A2, arrows, blue). (B) Showing a merged image of b-CTB (B1, arrows, red) co-labeled with ChAT (B2, arrows, blue). (C) Showing a merged image of FG (C1)-labeled neurons to be absent when stained doubly with GAD67 (C2, arrows, blue). (D) Showing a merged image of b-CTB (D1, arrows, red) co-labeled with GAD67 (D2, arrows, blue). Scale bars = 20 μm.
(DOC)

S1 License A production licence (ID: SCXK2009-0011) of experimental animal. The licence is provided to the Experimental Animal Center of Sun Yat-sen University by Guangdong Provincial Department of Science and Technilogy, in China.
(DOC)

S2 License A utilization licence (ID: SYXK2007-0081) of experimental animal. The licence is provided to the Experimental Animal Center of Sun Yat-sen University by Guangdong Provincial Department of Science and Technilogy, in China.
(DOC)
