# Supplementary material for: Cholera Toxin B Subunit Shows Transneuronal Tracing after Injection in an Injured Sciatic Nerve
Source: PLoS One. 2015 Dec 7;10(12):e0144030. doi: 10.1371/journal.pone.0144030 (PMC4671609; doi:10.1371/journal.pone.0144030)
Supplement: S1 Fig — (A) Showing a merged image of FG (A1, arrows, yellow)-labeled neurons stained doubly with ChAT (A2, arrows, blue). (B) Showing a merged image of b-CTB (B1, arrows, red) co-labeled with ChAT (B2, arrows, blue). (C) Showing a merged image of FG (C1)-labeled neurons to be absent when stained doubly with GAD67 (C2, arrows, blue). (D) Showing a merged image of b-CTB (D1, arrows, red) co-labeled with GAD67 (D2, arrows, blue). Scale bars = 20 μm. (DOC) [file pone.0144030.s001.doc]

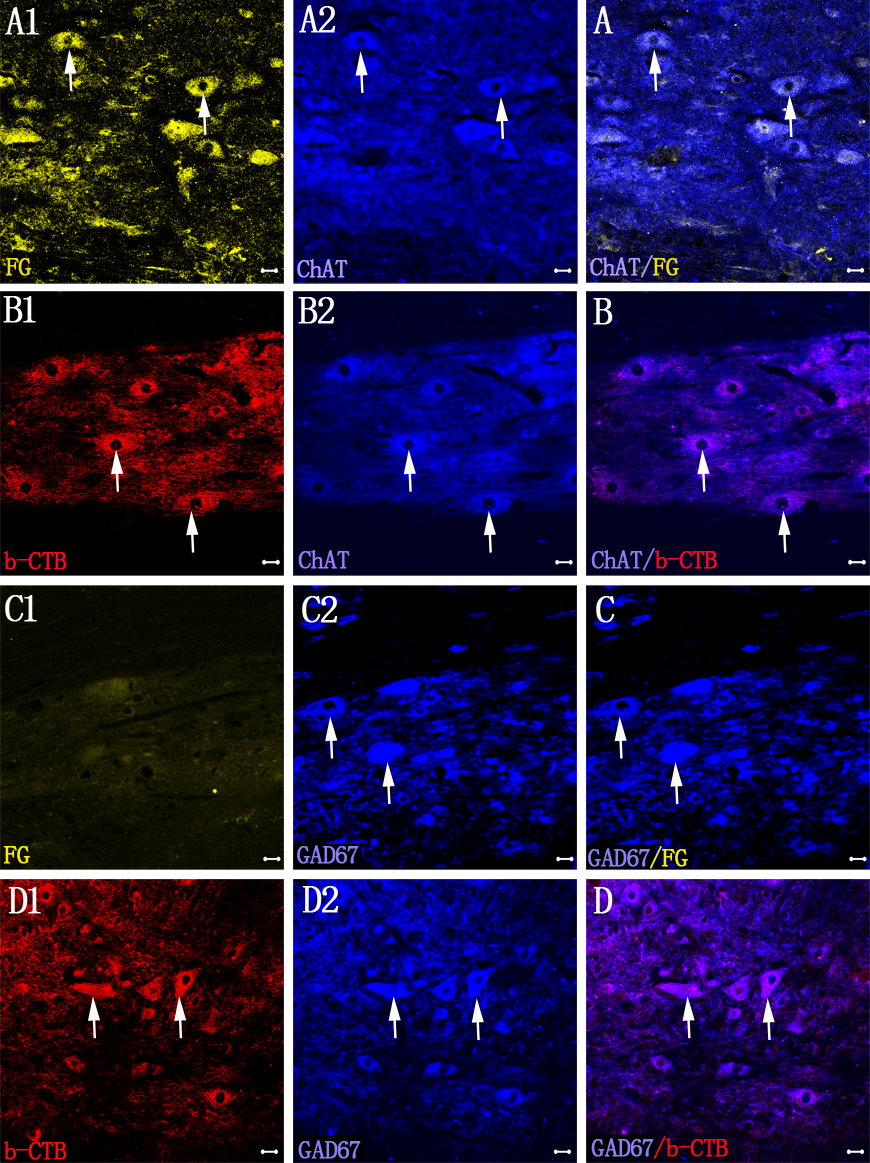


**S1 Fig. Comparison between b-CTB and FG tracing at L4 spinal segment.** (A) Showing a merged image of FG (A1, arrows, yellow)-labeled neurons stained doubly with ChAT (A2, arrows, blue). (B) Showing a merged image of b-CTB (B1, arrows, red) co-labeled with ChAT (B2, arrows, blue). (C) Showing a merged image of FG (C1)-labeled neurons to be absent when stained doubly with GAD67 (C2, arrows, blue). (D) Showing a merged image of b-CTB (D1, arrows, red) co-labeled with GAD67 (D2, arrows, blue). Scale bars = 20 µm.
