# Supplementary material for: Cholera Toxin B Subunit Shows Transneuronal Tracing after Injection in an Injured Sciatic Nerve
Source: PLoS One. 2015 Dec 7;10(12):e0144030. doi: 10.1371/journal.pone.0144030 (PMC4671609; doi:10.1371/journal.pone.0144030)
Supplement: S2 Fig — (A) Showing an overview of a transverse section of C7 spinal segment. Note the total absence of FG positive cells. (B) Map2-labeled staining (blue) helps to define the grey matter of spinal cord central area. (D) Showing a merged image of (A) and (B). Scale bars = 200 μm. (DOC) [file pone.0144030.s002.doc]

**
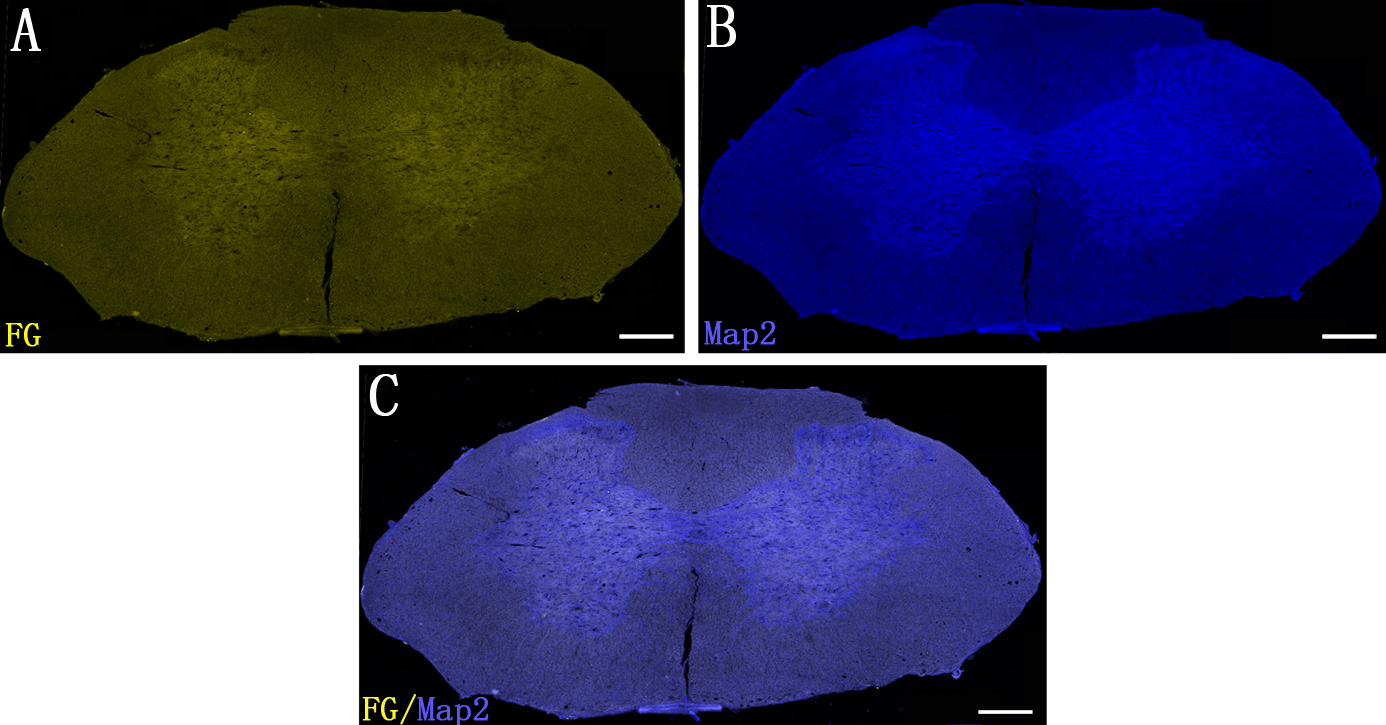
**

**S2 Fig. C7 spinal segment for FG tracing.** (A) Showing an overview of a transverse section of C7 spinal segment. Note the total absence of FG positive cells. (B) Map2-labeled staining (blue) helps to define the grey matter of spinal cord central area. (D) Showing a merged image of (A) and (B). Scale bars =200 µm.
