# Supplementary material for: Cholera Toxin B Subunit Shows Transneuronal Tracing after Injection in an Injured Sciatic Nerve
Source: PLoS One. 2015 Dec 7;10(12):e0144030. doi: 10.1371/journal.pone.0144030 (PMC4671609; doi:10.1371/journal.pone.0144030)
Supplement: S2 License — The licence is provided to the Experimental Animal Center of Sun Yat-sen University by Guangdong Provincial Department of Science and Technilogy, in China. (DOC) [file pone.0144030.s005.doc]

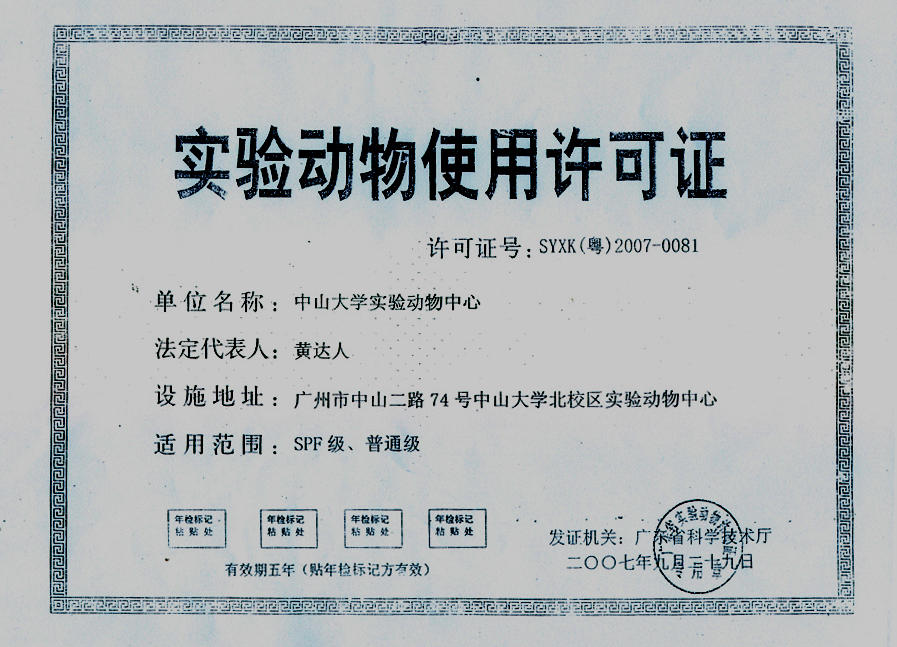


**S2 License. A utilization licence (ID: SYXK2007-0081) of experimental animal.** The licence is provided to the Experimental Animal Center of Sun Yat-sen University by Guangdong Provincial Department of Science and Technilogy, in China.
